# Willow Catkin Optimization Algorithm Applied in the TDOA-FDOA Joint Location Problem

**DOI:** 10.3390/e25010171

**Published:** 2023-01-14

**Authors:** Jeng-Shyang Pan, Si-Qi Zhang, Shu-Chuan Chu, Hong-Mei Yang, Bin Yan

**Affiliations:** 1 College of Computer Science and Engineering, Shandong University of Science and Technology, Qingdao 266590, China; 2 Department of Information Management, Chaoyang University of Technology, Taichung 41349, Taiwan; 3 College of Electronics, Communication and Physics, Shandong University of Science and Technology, Qingdao 266590, China

**Keywords:** Willow Catkin Optimization, metaheuristic optimization algorithm, CEC2017, TDOA-FDOA location problem, WSNs

## Abstract

The heuristic optimization algorithm is a popular optimization method for solving optimization problems. A novel meta-heuristic algorithm was proposed in this paper, which is called the Willow Catkin Optimization (WCO) algorithm. It mainly consists of two processes: spreading seeds and aggregating seeds. In the first process, WCO tries to make the seeds explore the solution space to find the local optimal solutions. In the second process, it works to develop each optimal local solution and find the optimal global solution. In the experimental section, the performance of WCO is tested with 30 test functions from CEC 2017. WCO was applied in the Time Difference of Arrival and Frequency Difference of Arrival (TDOA-FDOA) co-localization problem of moving nodes in Wireless Sensor Networks (WSNs). Experimental results show the performance and applicability of the WCO algorithm.

## 1. Introduction

The optimization problem [1] comes from the human pursuit of optimal results, and the traditional optimization methods mainly include the analytical and iterative methods. The theory and optimization algorithm gradually formed since the French mathematician Charles Cauchy proposed the most rapid descent method. With the emergence of complex, non-trivial and large-scale optimization problems, the solution of optimization problems has developed from Newton’s method, the conjugate gradient method and Powell’s method to intelligent optimization algorithms.

**Figure 1:**
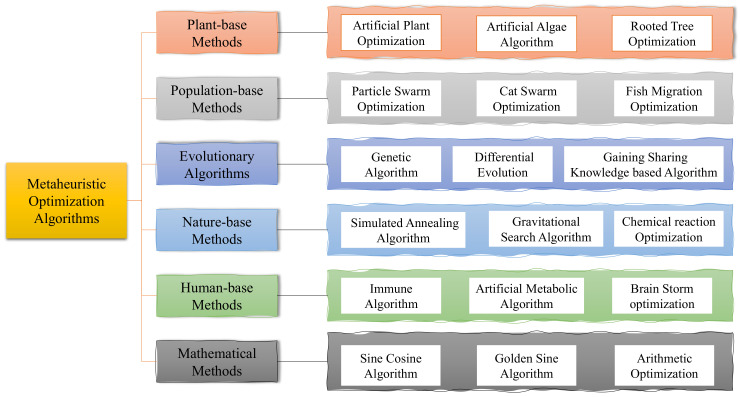
Meta-heuristic algorithm classification.

In recent decades, various meta-heuristic optimization algorithms [2,3] are proposed. They can be divided into six main categories in Figure 1: plant-based methods, population-based methods, evolutionary algorithms, nature-based methods, human-based methods, and mathematical methods. Most traditional intelligent optimization algorithms belong to the first five categories, which are inspired by the natural behaviors and natural phenomena of plants and animals in nature, by summarizing natural laws, discovering features, building models, adjusting parameters, and designing optimization algorithms based on natural laws to optimize specific problems. Plant-based methods find the global optimum by simulating the growth process of plants. The representative algorithms are Artificial Plant Optimization (APO) [4], the Artificial Algae Algorithm (AAA) [5], Rooted Tree Optimization (RTO) [6], and the Flower Pollination Algorithm (FPA) [7]. Population-based methods include Particle Swarm Optimization (PSO) [8], Cat Swarm Optimization (CSO) [9], Ant Colony Optimization (ACO) [10] and Fish Migration Optimization (FMO) [11]. There is also the Phasmatodea Population Evolution (PPE) [12] algorithm, which was recently proposed. This algorithm has multiple individuals, and the performance of the algorithm is affected by the initial values. Each individual in a population-based algorithm works independently or cooperatively to find the global optimum. Evolutionary-based algorithms include the Genetic Algorithm (GA) [13], Differential Evolution (DE) [14], and the Gaining Sharing Knowledge-based Algorithm (GSK) [15]. Such algorithms improve the ability to find the global optimum by continuously accumulating high-quality solutions. Nature-based methods include the Simulated Annealing Algorithm (SAA) [16], the Gravitational Search Algorithm (GSA) [17], and Chemical Reaction Optimization (CRO) [18]. These algorithms are designed by simulating the phenomena existing in nature and summarizing the objective laws of the phenomena to build an algorithmic model. Human-based algorithms include Immune Algorithm (IA) [19], Population Migration Algorithm (PMA) [20] and Brain Storm Optimization (BSO) [21]. Mathematical-based methods include the Sine Cosine Algorithm (SCA) [22], Golden Sine Algorithm (GSA) [23] and Arithmetic Optimization Algorithm (AOA) [24]. Many excellent intelligent optimization algorithms [25] have been proposed. Meta-heuristic optimization algorithms are cross-integrated with image processing, fault detection, path planning, particle filtering, feature selection, production scheduling, intrusion detection, support vector machines, wireless sensors, neural networks, and other technical fields for a wider range of applications. However, the fact that an algorithm performs well in optimizing a specific problem does not guarantee its effectiveness in other problems. No optimization algorithm can solve all optimization problems, which is the famous “No Free Lunch (NFL)” theory [26]. Therefore, researchers continue to improving existing algorithms and propose new ones to solve optimization problems in different fields.

Based on the NFL, a novel meta-heuristic algorithm was proposed in this paper, which is called the Willow Catkin Optimization algorithm. This algorithm was inspired by willow trees’ process of seed dispersal. Willow catkins are the seed of the willow tree. It is characterized by its ability to float to distant places with the help of the wind. Even a fragile wind will make it float with the wind, and throughout the floating process, it can float down to the land suitable for growth and take root and grow. In addition, willow catkins stick to each other and often gather in a cluster. Ultimately, the willow will always find a suitable place to take root. Based on the above characteristics, we divide the willow flocking algorithm into two processes to implement: fluttering with the wind and gathering into a cluster. We select CEC2017 [27] as the benchmark function set to test the effect of the WCO algorithm on the numerical function. Its results are compared in the three dimensions of 10D, 30D, and 50D with PSO, SCA, the Bat Algorithm (BA) [28], Bamboo Forest Growth Optimizer (BFGO) [29], Rafflesia Optimization Algorithm (ROA), and Tumbleweed Algorithm (TA) [30]. In addition, WCO was applied to the motion node localization problem in WSN to test the ability of the new algorithm to handle the practical problem. The WCO algorithm has achieved good results in this application compared with other algorithms.

Wireless sensor networks (WSN) [31] consist of many low-cost sensor nodes with communication and data processing capabilities. The current wireless sensor localization methods are divided into two types: non-ranging and ranging. Non-ranging-based localization methods do not require known distances, angles, or signal strengths and have the advantage of low hardware overhead, simple configuration, and high system scalability. Typical representatives of non-ranging localization algorithms include the distance vector-hop (DV-hop) [32,33] and multidimensional scaling maximum a posteriori probability estimation (MDS-MAP) [34,35]. Range-based methods extract measurement information based on distance, angle, etc. from different features of the radio signal, such as time of arrival (TOA) [36,37], Time Difference of Arrival (TDOA) [38,39], angle of arrival (AOA) [40] and radio signal strength indication (RSSI) [41,42], etc. TDOA has high positioning accuracy. However, it is also prone to time difference blurring, it is not easy to locate the target signal with high frequency, and the speed of the target cannot be determined. Adding Doppler frequency difference information to TDOA can improve the localization accuracy, eliminate the problem of time difference blurring, and determine the target’s speed. Many scholars have put forward their views on motion target localization techniques in recent years. The multi-station TDOA/FDOA co-localization [43,44] method is used to solve the nonlinear system of equations with the time difference and frequency difference, which has the defects of high complexity and extensive computation [45,46]. If the algorithm needs to be optimized enough, the localization results easily fall into the local optimum and slow convergence speed. Therefore, this paper applies the WCO algorithm to the motion node localization problem in WSN to optimize the joint TDOA/FDOA joint localization accuracy and improve the localization speed to reduce the time.

The remaining sections of this paper are arranged as follows. Section 2 will briefly introduce the formula principle of the iterative search of the WCO algorithm. Section 3 analyzes in detail the optimization results of the algorithm under the benchmark function test. Section 4 discusses the algorithm’s performance applied to the motion node localization problem in WSN. Finally, Section 5 gives the work of this paper and proposes future work directions.

## 2. Willow Catkin Optimization Algorithm

In this section, WCO is proposed as the new metaheuristic optimization algorithm. A hybrid exploration–exploitation model is proposed in WCO. The particles are divided into two parts in the search process, and exploration and exploitation are started simultaneously. The particles in different modes will have different behaviors and parameters.

### 2.1. Initialization Phase

In the initialization phase, several random seeds are generated from a willow tree and distributed in the solution space. The population is represented using a matrix with *N* rows and *D* columns. *N* is the number of particles in the population and *D* is the data dimension of each particle. The WCO starts with a random particle swarm and uses Equation (1) to generate random particles.

(1) $$x_{i}=r\times \left(UB-LB\right)+LB,i=1,2,\ldots ,N$$

*r* is a random number in the interval [0,1]. $x_{i}$ represents a solution of *D* dimensions. The upper and lower bounds of the solution space are UB and LB.

### 2.2. Search Phase

**Figure 2:**
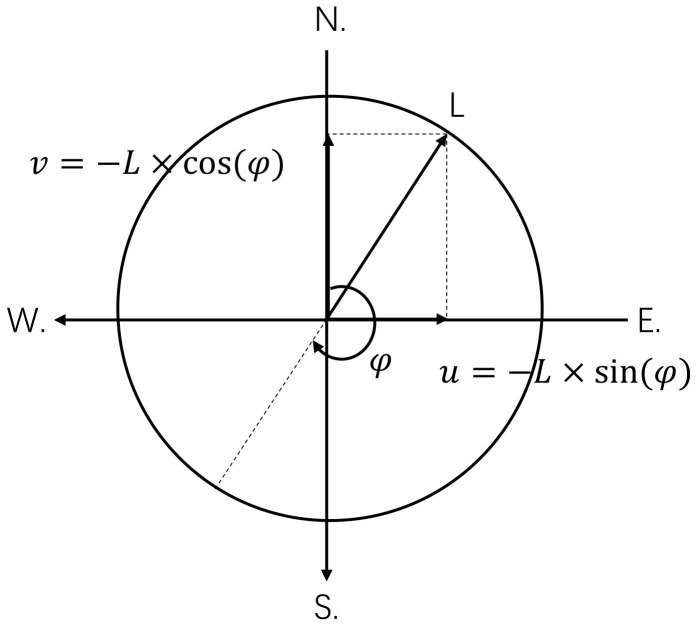
Obtaining *u* and *v* from wind vectors.

During this phase, a willow catkin falls from the willow trees and flutters in the wind, which are each affected by two parameters: wind direction and wind speed. Airflow in the atmosphere has both speed and direction. The wind is represented mathematically by a vector. Therefore, converting the meteorological wind vector into a “math” wind direction is necessary. It is common practice in meteorology to work with the wind’s *u* and *v* components. If the wind speed and direction have been measured, the component vectors of the wind, *u* and *v*, can be obtained as follows:

(2) $$\left\{\begin{array}{c} v=-ws\times \cos(wd)\\ u=-ws\times \sin(wd) \end{array}\right.$$

ws is the wind speed, and wd is the wind direction. Figure 2 shows the conversion of wind speed and direction into *u*, *v* components on a two-dimensional plane. In Figure 2, the symbol (N.) is an abbreviation for north.

After decomposing the wind speed and direction to obtain u,v, the particles can be updated. The particle update is mainly related to the wind direction and speed. The particle update is performed in the exploration phase using Equation (3).

(3) $$x_{i+1}=x_{i}+a\times \left(u\times v\right)+\left(2-a\right)\left(P_{g}-x_{i}\right)$$

where $x_{i}$ represents the individual’s current position and *a* is the variable that controls the shift from exploration to exploitation during the individual iteration. $P_{g}$ is the global optimum under the current iteration. *a* will change over the course of iterations, with the aim of balancing exploration and exploitation:

(4) $$a=c\times e^{-{\left(\frac{t}{1000}\right)}^{2}}$$

**Figure 3:**
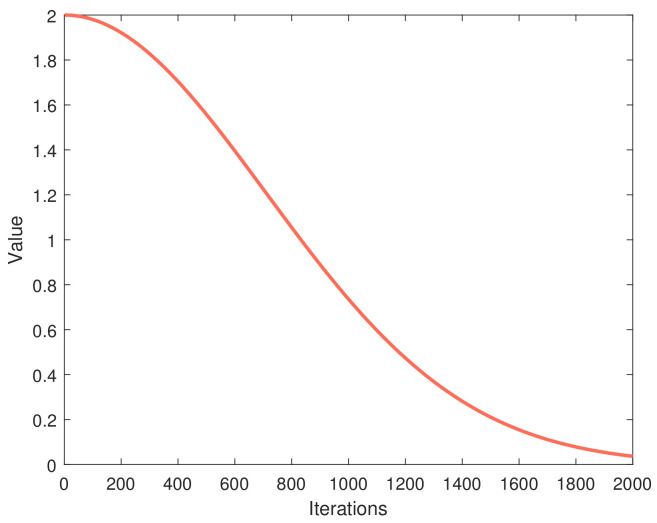
The function curve of parameter *a*.

*T* is the maximum number of iterations. *c* is a constant with value 2. *t* is the current number of iterations. As shown in Figure 3, when t<0.4T, a is greater than 1, the particle updates will be more influenced by the wind, making the particle positions more random.

In the iterative update of individuals, individuals that are too close together will be brought closer by the wind blowing, causing the two willow fuzzes to stick together. The distance di between the current individual and the global optimum is calculated. According to the easy adhesion radius *R*, if di>R, the two seeds are less likely to stick together. The random wind custom and wind direction are obtained by executing Equation (3). If di<=R, it means that the two seeds are likely to stick together and then execute Equation (8).

(5) $$\left\{\begin{array}{c} ws=r\times R\\ wd=r\times 2\pi \end{array}\right.$$

The $d_{i}$ is the distance of the particle from the global optimal solution. When $d_{i}>R$, ws is generated by Equation (5). *r* is a random number between 0 and 1, and *R* is the adhesion radius of willow catkins. Bringing the randomly generated ws and wd into Equation (2) can determine the individuals’ direction and distance of movement, so that each individual with a distance more than R will move randomly, improve the exploration ability of the algorithm and avoid the algorithm falling into local optimum.

(6) $$DW=1-\frac{\left|p_{g}-x_{i}\right|}{∥x_{i}-p_{g}∥}$$

(7) $$K=\frac{DW}{\sum_{i=1}^{D}DW_{i}}$$

(8) $$\left\{\begin{array}{ccc} ws & = & \mu \times \left(\sum_{i=1}^{D}K_{i}\left|p_{g}-x_{i}\right|\right)+\left(1-\mu \right)\times r_{2}\times R\\ wd & = & \arccos\left(\frac{x_{i}\cdot p_{g}}{∥x_{i}∥\times ∥p_{g}∥}\right)+r_{3}\times \frac{\pi }{8} \end{array}\right.$$

When $d_{i}<=R$, the weights are calculated by Equation (6) and Equation (7), and the wind speed and wind direction are calculated by Equation (8). ||·|| denotes the Euclidean distance between $x_{i}$ and $p_{g}$, and DW denotes the weight of each dimensional distance in $p_{g}$ and $x_{i}$ to the total distance. Equation (8) normalizes DW. μ is a random number from 0.4 to 0.6. $r_{2}$ and $r_{3}$ are random numbers in the interval [0, 1].

### 2.3. WCO Pseudo-Code

**Figure 4:**
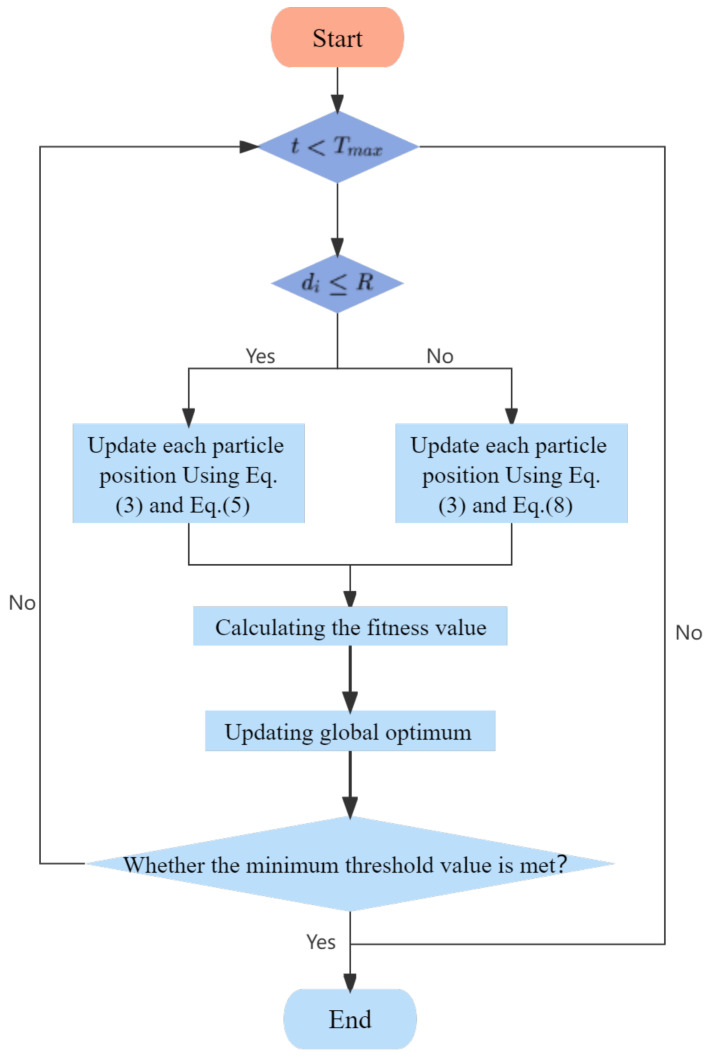
Flowchart of WCO.

The entire optimization process of the WCO algorithm starts with generating a series of random solutions. In each iteration, all individuals are adjusted according to the updated formula provided by WCO. The parameters control the development and exploration phase of the algorithm. The focus on exploration in the first half of the iteration makes the individuals move more randomly and explore more fully in the solution space. Exploitation is performed in the second half of the iteration to make the particles move closer to the global optimum. The attempt to find the optimal global solution is repeated throughout the population update until the maximum number of iterations is reached or an approximate global optimal solution is obtained. Algorithm  1 is the pseudo-code of WCO, and the flowchart is shown in Figure 4.
**Algorithm 1** WCO**Require:** Population size *N*, Max iteration *T*, Fitness function, Dimension *D*, Upper and Lower bounds UB,LB**Ensure:** Global best value GlobalBest1:Initialize the Pop, Gbest and GlobalBestPos of each group2:**while** (t<MaxIteration or met the mimimum threshold value) **do**3:    *a* = Equation (4)4:    **for** i=1:N **do**5:        **if** $R_{i}>R$ **then**6:           Generate ws and wd using Equation (5)7:        **else**8:           Generate ws and wd using Equations (6)–(8)9:        **end if**10:       Update Pop using Equations (2) and (3)11:       Calculate fitness value of population12:       Update GlobalBest and GlobalFmin13:    **end for**14:**end while**

### 2.4. Complexity Analysis

The complexity of WCO consists of three main components: initialization phase, search phase and fitness value calculation. The initialization phase mainly generates the population matrix of (N∗D), and the complexity of this phase is O(ND). The complexity of the search phase is O(TND). The positions of *N* individuals need to be updated in each iteration. Based on the above analysis, the time complexity of WCO is O(TND).

## 3. Experiment and Analysis

In this section, the CEC2017 benchmark is used to test the optimization performance of the WCO algorithm. The WCO is compared with other swarm intelligence optimization algorithms.

### 3.1. Parameter Settings

WCO experiments were performed using a PC with Windows 11 Professional 64-bit, Intel(R) Core (TM) i7-12700 CPU @ 2.10 GHz, 32.0 GB RAM, and Matlab R2021b. The WCO algorithm is run 30 times on each benchmark function, and the individual dimensions are 10, 30, and 50 dimensions. The upper and lower bounds are [−100, 100]. The results are compared with typical swarm intelligence optimization algorithms such as PSO, BA, SCA, BFGO, ROA, and TA. Table 1 shows the parameters of the comparison algorithm.

### 3.2. CEC2017 Benchmark Analysis

In CEC2017, there are 30 benchmark functions divided into four categories: unimodal, multimodal, hybrid, and composite. Unimodal functions (F1–F3) have only one global optimum and are used to compare the development capabilities of optimization algorithms. Simple multimodal functions (F4–F10) have many local optima, and the second better local optimum is far from the global optimum. In the hybrid functions (F11–F20), a hybrid function is composed of several essential functions. It comprises the unimodal function, multimodal function, and other essential functions. Hybrid functions test the performance of optimization algorithms on real-world optimization problems. The composition function (F21–F30) better merges the subfunctions properties and maintains continuity around the global/local optima. The local optimum, which has the smallest bias value, is the global optimum.

### 3.3. Statistical Results

The test results of each algorithm on each function are compared with those of the WCO algorithm. The symbol (<) indicates that the algorithm performs worse than the WCO algorithm in the current function. The symbol (>) indicates that the WCO algorithm performs poorly. The symbol (=) indicates that the two algorithms perform similarly on the current benchmark function. The comparison results of all benchmark functions are summarized in the last row of the table. The optimal solution in the table is bolded.

Table 2 shows the test results of the WCO algorithm in 10 dimensions. WCO outperforms BA, SCA, ROA, and TA by at least 25 benchmark functions, respectively. It outperforms the 24 benchmark functions of BFGO. It outperforms PSO 30% of the tested functions in terms of multimodal and composition functions. The most global optima were achieved in simple multimodal functions and composition functions, where 50% of the optima were obtained for 20 functions.

Table 3 shows that the WCO algorithm achieves 12 global optima in 30 dimensions, with PSO following closely behind with ten global optima. The algorithm also achieves four optimal solutions out of seven multimodal functions. The algorithm achieves 50% of the optimal values in 10 composition functions.

The test results in particle 50 dimensions are shown in Table 4. WCO achieves 60% optimal results on hybrid functions and 50% optimal values on compositions functions, indicating that WCO can still rely on the algorithm’s exploration and development capabilities to find the global optimal solution in the high-dimensional case. WCO outperforms PSO by 17 experimental results, while WCO is slightly weaker than PSO under unimodal functions but outperforms other comparative algorithms.

The above test results show that WCO handles multimodal functions and composition functions better under low-dimensional optimization functions and performs better for compound functions when optimizing high-dimensional problems. The conclusions of this experiment show that the WCO algorithm has excellent global exploration ability and can escape from the local optimal solution for better global search when the particles near the global optimal solution are trapped in the local optimal solution. During the gradual shift of the algorithm from the exploration phase to the development phase, the distance $d_{i}$ of the particles from the global optimal solution is less than *R*, so the particles keep the process of random exploration, which makes the algorithm have the ability to escape from the local optimal solution even in the late iteration.

## 4. WCO for TDOA and FDOA Joint Location

### Fitness function of TDOA and FDOA Joint Location

In the 3D spatial coordinate system, the motion node localization in WSN typically uses the TDOA-FDOA joint localization method. It is used to determine the position and velocity of the localized target by receiving TDOA information and FDOA information between two independent receivers. Suppose the position and velocity of the target are $u={\left[x,y,z\right]}^{T}$, $\dot{u}={\left[\dot{x},\dot{y},\dot{z}\right]}^{T}$. The coordinates and velocity of each base station are $s_{i}={\left[x_{i},y_{i},z_{i}\right]}^{T}$ and $\dot{s_{i}}={\left[\dot{x_{i}},\dot{y_{i}},\dot{z_{i}}\right]}^{T}$, i=1,2,…,M, where ${\left(\right)}^{\circ }$ represents the true value of the zero error. *M* is the number of anchor nodes. M≥4 is required for positioning in 3D space. Anchor nodes cannot be in the same plane or line in the 3D spatial coordinate system.

Usually, the first anchor node is chosen as a reference. The distance between the target and the anchor node *i* is $R_{i}$.

(9) $$R_{i}^{\circ }=∥U-X_{i}∥=\sqrt{{\left(x-x_{i}\right)}^{2}+{\left(y-y_{i}\right)}^{2}+{\left(z-z_{i}\right)}^{2}}$$

The distance difference between the target node to anchor node *i* and anchor node 1 is $R_{i1}$.

(10) $$R_{i1}=R_{i}^{\circ }-R_{1}^{\circ }+\eta_{i1}=c\times t_{i1}+\eta_{i1}\left(i=2,3,\ldots ,M\right)$$

where *c* is the speed of light, and $t_{i1}$ is the time difference between the arrival of the signal from the target node to the anchor node *i* and the anchor node 1. $\eta_{i1}$ is the distance noise error between the observation $R_{i}$ and $R_{1}$. Differentiating the time in Equation (10) yields the Doppler frequency difference observation equation:

(11) $${\dot{R}}_{i1}={\dot{R}}_{i}^{\circ }-{\dot{R}}_{1}^{\circ }+{\dot{\eta }}_{i1}=\frac{cf_{i1}}{f_{c}}+{\dot{\eta }}_{i1}\left(i=2,3,...,M\right)$$

$f_{i1}$ is the Doppler frequency difference. $f_{c}$ is the carrier frequency. ${\dot{R}}_{i}$ is the rate of change of $R_{i}$ with time. ${\dot{\eta }}_{i1}$ is the distance noise error between the observation ${\dot{R}}_{i}$ and ${\dot{R}}_{1}$. The differentiation of time in Equation (9) yields ${\dot{R}}_{i}$:

(12) $${\dot{R}}_{i}=\frac{{\left(\dot{U}-{\dot{X}}_{i}\right)}^{T}\left(U-X_{i}\right)}{R_{i}}$$

We shift the term for Equation (10) and square both sides of the equal sign.

(13) $$\begin{array}{cc} R_{i1}^{2}+2R_{i1}R_{1}^{\circ } & =R_{i}^{\circ 2}-R_{1}^{\circ 2}+\eta_{i}\\ \; & =X_{i}^{T}X_{i}-X_{1}^{T}X_{1}-2{\left(X_{i}-X_{1}\right)}^{T}U+\eta_{i1} \end{array}$$

where the noise error term in Equation (13) can be expressed as:

(14) $$\varepsilon_{i1}=R_{i1}^{2}+2R_{i1}R_{1}-X_{i}^{T}X_{i}+X_{1}^{T}X_{1}+2{\left(X_{i}-X_{1}\right)}^{T}U$$

Expressing Equation (14) in matrix form yields:

(15) $$\varepsilon_{t}=h_{t}-G_{t}\theta$$

(16) $$\varepsilon_{t}={\left[\begin{array}{c} \varepsilon_{t2},\varepsilon_{t3},\ldots ,\varepsilon_{tM} \end{array}\right]}^{T}$$

(17) $$h_{t}=\left[\begin{array}{c} R_{21}^{2}-X_{2}^{T}X_{2}+X_{1}^{T}X_{1}\\ R_{31}^{2}-X_{3}^{T}X_{3}+X_{1}^{T}X_{1}\\ \vdots \\ R_{i1}^{2}-X_{i}^{T}X_{i}+X_{1}^{T}X_{1} \end{array}\right]$$

(18) $$G_{t}=-2\left[\begin{array}{ccccc} {\left(X_{2}-X_{1}\right)}^{T} & R_{21}^{2} & 0^{T} & 0 & \\ {\left(X_{3}-X_{1}\right)}^{T} & R_{31}^{2} & 0^{T} & 0 & \\ \vdots \\ {\left(X_{M}-X_{1}\right)}^{T} & R_{M1}^{2} & 0^{T} & 0 \end{array}\right]$$

(19) $$\theta ={\left[\begin{array}{c} U,R_{1},\dot{U},{\dot{R}}_{1} \end{array}\right]}^{T}$$

In the above equation, $0^{T}$ is a three-dimensional row vector consisting of zeros. θ is obtained by the optimization algorithm and is an eight-dimensional column vector. The goal of the algorithm is to find the global optimal solution that makes the TDOA localization error $\varepsilon_{t}$ minimum, and this solution is the position of the target.

Equation (13) is a nonlinear equation containing the position of the target node, which can only calculate the target position because it only contains the time difference information. By introducing FDOA information combined with TDOA, not only can the instantaneous velocity information of the target be solved, but also a higher position accuracy than that of TDOA localization alone can be obtained. Thus, differentiating the time of Equation (13) yields the following:

(20) $$R_{i1}{\dot{R}}_{i1}+{\dot{R}}_{i1}R_{1}^{\circ }+R_{i1}{\dot{R}}_{1}^{\circ }={\dot{X}}_{i}^{T}X_{i}-{\dot{X}}_{1}^{T}X_{1}-{\left({\dot{X}}_{i}^{T}-{\dot{X}}_{1}\right)}^{T}U+{\left(X_{i}^{T}-X_{1}\right)}^{T}\dot{U}+{\dot{\eta }}_{i1}$$

Expressing Equation (20) in matrix form.

(21) $$\varepsilon_{f}=h_{f}-G_{f}\theta$$

(22) $$\varepsilon_{f}={\left[\begin{array}{c} \varepsilon_{f2},\varepsilon_{f3},...,\varepsilon_{fM} \end{array}\right]}^{T}$$

(23) $$h_{f}=2\left[\begin{array}{c} R_{21}{\dot{R}}_{21}-{\dot{X}}_{2}^{T}X_{2}+{\dot{X}}_{1}^{T}X_{1}\\ R_{31}{\dot{R}}_{31}-{\dot{X}}_{3}^{T}X_{3}+{\dot{X}}_{1}^{T}X_{1}\\ \vdots \\ R_{i1}{\dot{R}}_{M1}-{\dot{X}}_{i}^{T}X_{i}+{\dot{X}}_{1}^{T}X_{1} \end{array}\right]$$

(24) $$G_{f}=-2\left[\begin{array}{ccccc} {\left({\dot{X}}_{2}-{\dot{X}}_{1}\right)}^{T} & {\dot{R}}_{21} & {\left(X_{2}-X_{1}\right)}^{T} & R_{21} & \\ {\left({\dot{X}}_{3}-{\dot{X}}_{1}\right)}^{T} & {\dot{R}}_{31} & {\left(X_{3}-X_{1}\right)}^{T} & R_{31} & \\ \vdots \\ {\left({\dot{X}}_{M}-{\dot{X}}_{1}\right)}^{T} & {\dot{R}}_{M1} & {\left(X_{M}-X_{1}\right)}^{T} & R_{M1} \end{array}\right]$$

The joint TDOA-FDOA error matrix can be obtained from Equations (15) and (21) as follows:

(25) $$\varepsilon =\left[\begin{array}{c} \varepsilon_{t}\\ \varepsilon_{f} \end{array}\right]=h-G\theta ,h=\left[\begin{array}{c} h_{t}\\ h_{f} \end{array}\right],g=\left[\begin{array}{c} g_{t}\\ g_{f} \end{array}\right]$$

The above analysis finally leads to the fitness function:

(26) $$fitness=∥h-G\theta ∥$$

In order to realize the reconnaissance of radiation source by space platform and verify the proposed algorithm’s effectiveness, this experiment will use five base stations to complete the target localization, whose coordinate positions and velocities are shown in Table 5. WCO is compared with PSO, BA, and TSWLS through 1000 Monte Carlo simulation experiments, and the simulation results are specifically analyzed. The standard parameters used in the experiments include the number of particles N=100, T=500, and D=6. The time difference and the Doppler frequency difference are independent in this simulation environment. The estimated value is the actual value plus the Gaussian white noise with the mean value of 0. The noise range is from −20 to 20 dB.

In this experiment, root mean square error (RMSE) and bias will be used to analyze the positioning accuracy of the position and velocity of the target, which is calculated as follows:

(27) $$\begin{array}{cc} RMSE(u) & =\sqrt{\frac{\sum_{i=1}^{L}∥u-u^{\circ }∥}{L}}\\ RMSE(\dot{u}) & =\sqrt{\frac{\sum_{i=1}^{L}∥\dot{u}-{\dot{u}}^{\circ }∥}{L}}\\ Bias(u) & =\frac{∥\sum_{i=1}^{L}u-u^{\circ }\;∥}{L}\\ Bias(\dot{u}) & =\frac{∥\sum_{i=1}^{L}\dot{u}-{\dot{u}}^{\circ }∥}{L} \end{array}$$

**Figure 5:**
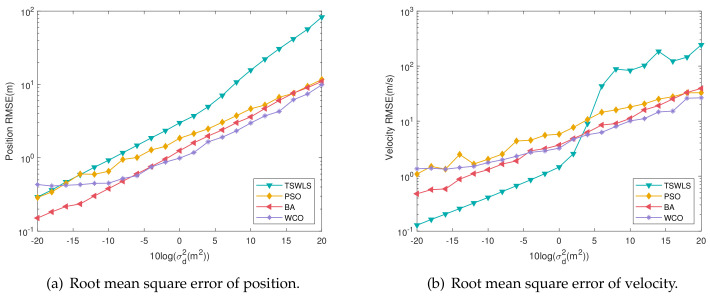
Root mean square error of each algorithm.

**Figure 6:**
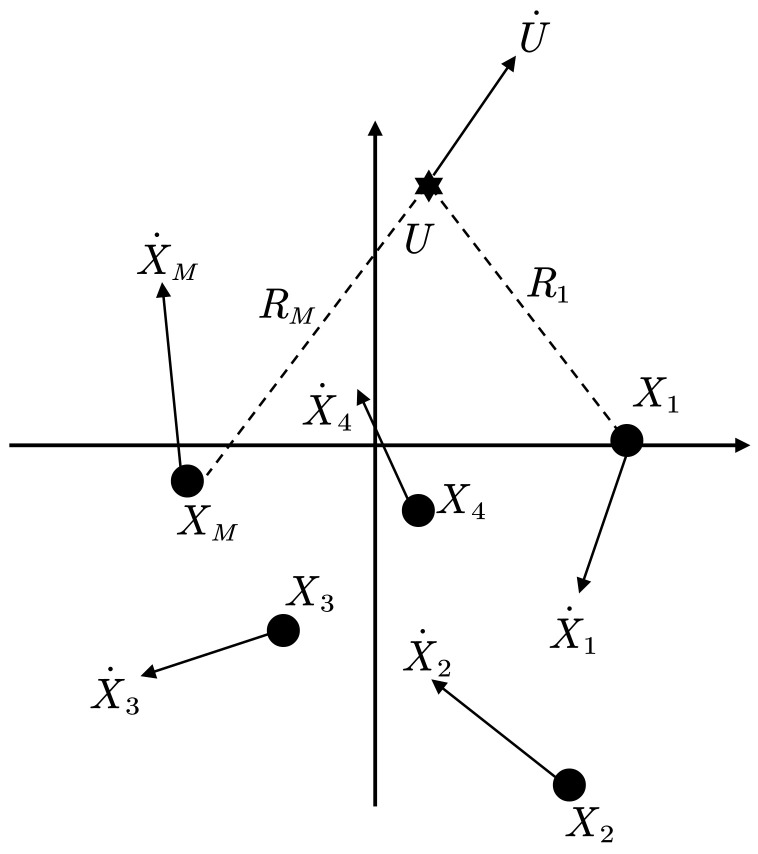
TDOA/FDOA Joint Location Model.

Figure 5 shows the positioning accuracy analysis of the target position and velocity under the conditions of Figure 6; the actual position of the target is [285, 325, 275] m and the velocity is [−20, 15, 40] m/s. From the above two figures, it can be seen that the position deviation of WCO is more significant than that of the BA algorithm when the noise is lower than −5 dB, and the overall positioning accuracy advantage of WCO is reflected at 0–20 dB. The BA algorithm’s position deviation increases with the error and finally approaches PSO after 10 dB. Overall, PSO, BA and WCO are better than the TSWLS algorithm. Regarding velocity error, TSWLS is significantly better than the three algorithms before 2 dB. However, due to the characteristics of the TSWLS algorithm, the results are more susceptible to perturbation as the noise increases, and WCO achieves a better velocity error after 2 dB.

**Figure 7:**
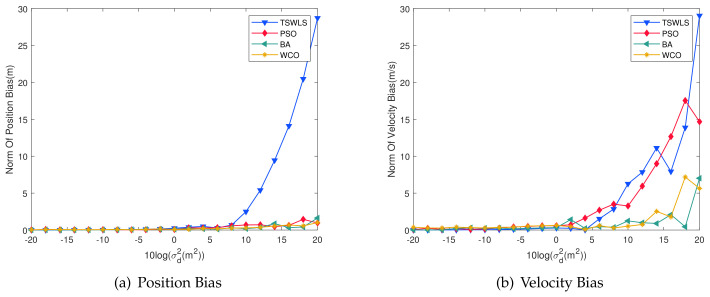
Bias of each algorithm.

Figure 7 shows that the position and velocity deviations remain basically the same for the three algorithms until 0 dB, and then, the positioning accuracy of WCO improves significantly in the position deviation. When the noise is greater than 5 dB and less than 12 dB, the speed measurement error of WCO is less than BA. Overall, the position deviation of WCO is better than the velocity deviation regardless of the position deviation or velocity deviation.

As shown in Table 6, the execution time required by the WCO algorithm is compared with TSWLS and the cluster intelligence algorithms of PSO and BA. It can be seen that the WCO algorithm takes the least amount of time to complete an estimation, reducing the running time by 18.6% compared to TSWLS, and improving the execution speed by 34.4% and 31.7% compared to PSO and BA, respectively.

## 5. Conclusions

By simulating the behavior of willow seeds falling with the wind, a new meta-heuristic optimization algorithm is proposed, which is called the Willow Catkin Optimization algorithm. In order to verify the performance of the algorithm, 30 benchmark functions from CEC2017 are used. In comparing the results of the WCO algorithm with other heuristic algorithms, it is concluded that the WCO algorithm is very competitive in dealing with optimization problems. Finally, WCO is applied to the joint TDOA/FDOA localization problem of motion nodes in WSN. The results show that the localization and velocity accuracy of WCO is higher than the comparison algorithms. Regarding algorithm execution speed, the WCO algorithm outperforms TSWLS 18.6%, PSO 34.4% and BA 31.7%. Adding binary and multi-objective versions [47] and applying it to more practical applications [48,49] is a valuable direction for future research.

## Figures and Tables

**Table 1:** Parameter setting of the comparison algorithm.

Algorithm	Parameter	Value
	Vmax	10
PSO	$c_{1},c_{2}$	2
	*w*	0.2
	$r_{0}$	0.7
BA	α	0.9
	γ	0.9
SCA	*a*	2
BFGO	*Q*	2
	*A*	2.5
ROA	*f*	40
	*B*	0.1
	φ	−0.78545
TA	gc	50
	*t*	2

**Table 2:** The 10 Dim Simulation Results of CEC 2017 Benchmark Function.

Function	PSO	BA	SCA	BFGO	ROA	TA	WCO
F1	** $1.00\times 10^{2}$ **	$3.66\times 10^{5}$	$5.28\times 10^{8}$	$1.44\times 10^{4}$	$5.59\times 10^{3}$	$1.75\times 10^{4}$	$5.74\times 10^{2}$
F2	** $2.00\times 10^{2}$ **	** $2.00\times 10^{2}$ **	$8.52\times 10^{6}$	$3.69\times 10^{2}$	** $2.00\times 10^{2}$ **	$1.90\times 10^{3}$	** $2.00\times 10^{2}$ **
F3	** $3.00\times 10^{2}$ **	$3.01\times 10^{2}$	$9.41\times 10^{2}$	** $3.00\times 10^{2}$ **	** $3.00\times 10^{2}$ **	** $3.00\times 10^{2}$ **	** $3.00\times 10^{2}$ **
F4	** $4.00\times 10^{2}$ **	$4.01\times 10^{2}$	$4.42\times 10^{2}$	$4.05\times 10^{2}$	$4.02\times 10^{2}$	$4.05\times 10^{2}$	$4.01\times 10^{2}$
F5	$5.42\times 10^{2}$	$5.52\times 10^{2}$	$5.45\times 10^{2}$	$5.24\times 10^{2}$	$5.62\times 10^{2}$	$5.22\times 10^{2}$	** $5.21\times 10^{2}$ **
F6	$6.09\times 10^{2}$	$6.36\times 10^{2}$	$6.17\times 10^{2}$	$6.06\times 10^{2}$	$6.43\times 10^{2}$	$6.10\times 10^{2}$	** $6.03\times 10^{2}$ **
F7	** $7.18\times 10^{2}$ **	$8.49\times 10^{2}$	$7.69\times 10^{2}$	$7.24\times 10^{2}$	$7.54\times 10^{2}$	$7.53\times 10^{2}$	$7.28\times 10^{2}$
F8	$8.22\times 10^{2}$	$8.44\times 10^{2}$	$8.34\times 10^{2}$	$8.18\times 10^{2}$	$8.88\times 10^{2}$	$8.31\times 10^{2}$	** $8.17\times 10^{2}$ **
F9	$9.10\times 10^{2}$	$1.68\times 10^{3}$	$1.01\times 10^{3}$	$9.06\times 10^{2}$	$9.43\times 10^{2}$	$9.57\times 10^{2}$	** $9.00\times 10^{2}$ **
F10	$2.82\times 10^{3}$	$2.19\times 10^{3}$	$2.14\times 10^{3}$	$1.76\times 10^{3}$	$2.92\times 10^{3}$	$1.99\times 10^{3}$	** $1.58\times 10^{3}$ **
F11	** $1.12\times 10^{3}$ **	$1.21\times 10^{3}$	$1.18\times 10^{3}$	$1.16\times 10^{3}$	$1.16\times 10^{3}$	$1.17\times 10^{3}$	$1.14\times 10^{3}$
F12	** $2.44\times 10^{3}$ **	$4.41\times 10^{5}$	$6.51\times 10^{6}$	$5.27\times 10^{4}$	$5.36\times 10^{4}$	$2.34\times 10^{6}$	$5.28\times 10^{4}$
F13	** $1.88\times 10^{3}$ **	$2.21\times 10^{4}$	$1.60\times 10^{4}$	$1.26\times 10^{4}$	$4.90\times 10^{3}$	$1.09\times 10^{4}$	$1.25\times 10^{4}$
F14	** $1.43\times 10^{3}$ **	$1.96\times 10^{3}$	$1.57\times 10^{3}$	$1.46\times 10^{3}$	$4.20\times 10^{3}$	$1.49\times 10^{3}$	$1.49\times 10^{3}$
F15	** $1.60\times 10^{3}$ **	$6.84\times 10^{3}$	$1.96\times 10^{3}$	$1.79\times 10^{3}$	$2.12\times 10^{3}$	$1.92\times 10^{3}$	$1.66\times 10^{3}$
F16	$1.84\times 10^{3}$	$2.04\times 10^{3}$	$1.71\times 10^{3}$	$1.72\times 10^{3}$	$1.73\times 10^{3}$	$1.72\times 10^{3}$	** $1.68\times 10^{3}$ **
F17	$1.75\times 10^{3}$	$1.81\times 10^{3}$	$1.77\times 10^{3}$	$1.76\times 10^{3}$	$1.86\times 10^{3}$	$1.76\times 10^{3}$	** $1.72\times 10^{3}$ **
F18	$1.89\times 10^{3}$	$1.52\times 10^{4}$	$6.29\times 10^{4}$	$1.92\times 10^{4}$	$1.29\times 10^{4}$	$1.74\times 10^{4}$	** $1.52\times 10^{3}$ **
F19	** $1.90\times 10^{3}$ **	$3.49\times 10^{3}$	$2.68\times 10^{3}$	$1.96\times 10^{3}$	$2.01\times 10^{3}$	$2.04\times 10^{3}$	$1.94\times 10^{3}$
F20	$2.10\times 10^{3}$	$2.17\times 10^{3}$	$2.08\times 10^{3}$	$2.09\times 10^{3}$	$2.08\times 10^{3}$	$2.07\times 10^{3}$	** $2.06\times 10^{3}$ **
F21	$2.33\times 10^{3}$	$2.31\times 10^{3}$	$2.23\times 10^{3}$	$2.26\times 10^{3}$	$2.35\times 10^{3}$	$2.23\times 10^{3}$	** $2.20\times 10^{3}$ **
F22	$2.31\times 10^{3}$	$2.31\times 10^{3}$	$2.35\times 10^{3}$	$2.30\times 10^{3}$	$2.31\times 10^{3}$	$2.33\times 10^{3}$	** $2.27\times 10^{3}$ **
F23	$2.64\times 10^{3}$	$2.66\times 10^{3}$	$2.65\times 10^{3}$	$2.63\times 10^{3}$	** $2.62\times 10^{3}$ **	$2.63\times 10^{3}$	** $2.62\times 10^{3}$ **
F24	$2.76\times 10^{3}$	$2.84\times 10^{3}$	$2.76\times 10^{3}$	$2.71\times 10^{3}$	$2.78\times 10^{3}$	$2.71\times 10^{3}$	** $2.58\times 10^{3}$ **
F25	** $2.90\times 10^{3}$ **	$2.93\times 10^{3}$	$2.95\times 10^{3}$	$2.91\times 10^{3}$	$2.95\times 10^{3}$	$2.93\times 10^{3}$	$2.92\times 10^{3}$
F26	$2.90\times 10^{3}$	$3.22\times 10^{3}$	$3.05\times 10^{3}$	$3.10\times 10^{3}$	$2.90\times 10^{3}$	$2.93\times 10^{3}$	** $2.88\times 10^{3}$ **
F27	$3.13\times 10^{3}$	$3.14\times 10^{3}$	$3.10\times 10^{3}$	** $3.08\times 10^{3}$ **	$3.10\times 10^{3}$	$3.10\times 10^{3}$	$3.10\times 10^{3}$
F28	** $3.20\times 10^{3}$ **	$3.25\times 10^{3}$	$3.27\times 10^{3}$	$3.26\times 10^{3}$	$3.41\times 10^{3}$	$3.21\times 10^{3}$	$3.23\times 10^{3}$
F29	** $3.19\times 10^{3}$ **	$3.32\times 10^{3}$	$3.22\times 10^{3}$	$3.26\times 10^{3}$	$3.20\times 10^{3}$	$3.23\times 10^{3}$	** $3.19\times 10^{3}$ **
F30	** $3.69\times 10^{3}$ **	$1.28\times 10^{4}$	$6.23\times 10^{5}$	$4.98\times 10^{3}$	$8.22\times 10^{5}$	$8.41\times 10^{5}$	$9.34\times 10^{3}$
</=/>	15/3/12	28/2/0	29/1/0	24/1/5	25/4/1	25/3/2	-

**Table 3:** The 30 Dim Simulation Results of CEC 2017 Benchmark Function.

Function	PSO	BA	SCA	BFGO	ROA	TA	WCO
F1	$5.69\times 10^{5}$	$7.02\times 10^{6}$	$1.44\times 10^{10}$	$8.72\times 10^{5}$	$1.93\times 10^{3}$	$7.83\times 10^{6}$	** $1.31\times 10^{5}$ **
F2	$3.43\times 10^{8}$	** $6.17\times 10^{3}$ **	$8.320\times 10^{35}$	$1.46\times 10^{20}$	$1.42\times 10^{8}$	$2.41\times 10^{22}$	$4.27\times 10^{12}$
F3	$3.29\times 10^{2}$	$3.74\times 10^{2}$	$4.90\times 10^{4}$	$1.32\times 10^{4}$	** $3.00\times 10^{2}$ **	$1.46\times 10^{4}$	$2.07\times 10^{4}$
F4	$5.17\times 10^{2}$	** $4.54\times 10^{2}$ **	$1.73\times 10^{3}$	$4.83\times 10^{2}$	$4.91\times 10^{2}$	$6.21\times 10^{2}$	$5.10\times 10^{2}$
F5	$6.49\times 10^{2}$	$8.64\times 10^{2}$	$7.93\times 10^{2}$	$6.66\times 10^{2}$	$7.91\times 10^{2}$	$6.92\times 10^{2}$	** $6.22\times 10^{2}$ **
F6	$6.38\times 10^{2}$	$6.74\times 10^{2}$	$6.56\times 10^{2}$	$6.36\times 10^{2}$	$6.67\times 10^{2}$	$6.47\times 10^{2}$	** $6.34\times 10^{2}$ **
F7	$8.86\times 10^{2}$	$1.71\times 10^{3}$	$1.15\times 10^{3}$	$8.70\times 10^{2}$	$1.02\times 10^{3}$	$1.08\times 10^{3}$	** $8.63\times 10^{2}$ **
F8	$9.49\times 10^{2}$	$1.12\times 10^{3}$	$1.07\times 10^{3}$	$9.37\times 10^{2}$	$1.06\times 10^{3}$	$9.76\times 10^{2}$	** $9.02\times 10^{2}$ **
F9	$2.87\times 10^{3}$	$1.41\times 10^{4}$	$6.03\times 10^{3}$	** $2.38\times 10^{3}$ **	$1.11\times 10^{4}$	$7.05\times 10^{3}$	$2.78\times 10^{3}$
F10	$5.86\times 10^{3}$	$5.85\times 10^{3}$	$8.44\times 10^{3}$	$5.26\times 10^{3}$	** $3.99\times 10^{3}$ **	$5.16\times 10^{3}$	$5.08\times 10^{3}$
F11	** $1.21\times 10^{3}$ **	$1.29\times 10^{3}$	$2.45\times 10^{3}$	$1.28\times 10^{3}$	$1.40\times 10^{3}$	$1.42\times 10^{3}$	$1.25\times 10^{3}$
F12	** $1.14\times 10^{5}$ **	$1.01\times 10^{7}$	$1.58\times 10^{9}$	$7.47\times 10^{6}$	$1.75\times 10^{5}$	$6.36\times 10^{7}$	$1.44\times 10^{7}$
F13	** $1.55\times 10^{4}$ **	$5.11\times 10^{5}$	$4.66\times 10^{8}$	$1.58\times 10^{5}$	$2.64\times 10^{4}$	$1.38\times 10^{5}$	$1.02\times 10^{5}$
F14	** $1.72\times 10^{3}$ **	$1.67\times 10^{4}$	$2.45\times 10^{5}$	$4.76\times 10^{4}$	$2.26\times 10^{4}$	$3.85\times 10^{4}$	$2.16\times 10^{4}$
F15	** $1.94\times 10^{3}$ **	$1.17\times 10^{5}$	$1.76\times 10^{7}$	$6.34\times 10^{4}$	$8.24\times 10^{4}$	$6.12\times 10^{4}$	$3.07\times 10^{4}$
F16	** $2.60\times 10^{3}$ **	$3.65\times 10^{3}$	$3.76\times 10^{3}$	$2.99\times 10^{3}$	$2.62\times 10^{3}$	$3.05\times 10^{3}$	$2.84\times 10^{3}$
F17	$2.17\times 10^{3}$	$2.88\times 10^{3}$	$2.57\times 10^{3}$	$2.30\times 10^{3}$	$2.55\times 10^{3}$	$2.29\times 10^{3}$	** $2.14\times 10^{3}$ **
F18	** $8.62\times 10^{3}$ **	$2.92\times 10^{5}$	$6.20\times 10^{6}$	$3.96\times 10^{5}$	$6.33\times 10^{4}$	$1.30\times 10^{6}$	$2.97\times 10^{5}$
F19	** $2.15\times 10^{3}$ **	$2.05\times 10^{6}$	$4.67\times 10^{7}$	$6.06\times 10^{4}$	$8.89\times 10^{4}$	$3.52\times 10^{6}$	$2.27\times 10^{6}$
F20	$2.72\times 10^{3}$	$2.85\times 10^{3}$	$2.72\times 10^{3}$	$2.62\times 10^{3}$	$3.01\times 10^{3}$	$2.56\times 10^{3}$	** $2.52\times 10^{3}$ **
F21	$2.46\times 10^{3}$	$2.63\times 10^{3}$	$2.57\times 10^{3}$	$2.48\times 10^{3}$	$2.67\times 10^{3}$	$2.48\times 10^{3}$	** $2.40\times 10^{3}$ **
F22	$7.45\times 10^{3}$	$6.57\times 10^{3}$	$8.72\times 10^{3}$	$4.92\times 10^{3}$	$7.56\times 10^{3}$	$5.27\times 10^{3}$	** $2.44\times 10^{3}$ **
F23	$3.02\times 10^{3}$	$3.33\times 10^{3}$	$3.02\times 10^{3}$	$2.92\times 10^{3}$	$3.03\times 10^{3}$	$2.90\times 10^{3}$	** $2.81\times 10^{3}$ **
F24	$3.08\times 10^{3}$	$3.46\times 10^{3}$	$3.19\times 10^{3}$	$3.14\times 10^{3}$	$3.04\times 10^{3}$	$3.08\times 10^{3}$	** $2.96\times 10^{3}$ **
F25	$2.90\times 10^{3}$	** $2.89\times 10^{3}$ **	$3.27\times 10^{3}$	$2.91\times 10^{3}$	** $2.89\times 10^{3}$ **	$2.97\times 10^{3}$	$2.92\times 10^{3}$
F26	** $2.81\times 10^{3}$ **	$9.28\times 10^{3}$	$7.25\times 10^{3}$	$6.42\times 10^{3}$	$6.60\times 10^{3}$	$6.45\times 10^{3}$	$4.19\times 10^{3}$
F27	$3.37\times 10^{3}$	$3.23\times 10^{3}$	$3.44\times 10^{3}$	** $3.20\times 10^{3}$ **	$3.34\times 10^{3}$	$3.43\times 10^{3}$	$3.35\times 10^{3}$
F28	$3.24\times 10^{3}$	$3.30\times 10^{3}$	$3.95\times 10^{3}$	$3.27\times 10^{3}$	** $3.22\times 10^{3}$ **	$3.34\times 10^{3}$	$3.25\times 10^{3}$
F29	$4.36\times 10^{3}$	$4.89\times 10^{3}$	$4.87\times 10^{3}$	$4.37\times 10^{3}$	$5.04\times 10^{3}$	$4.36\times 10^{3}$	** $4.07\times 10^{3}$ **
F30	** $2.36\times 10^{4}$ **	$3.36\times 10^{6}$	$1.05\times 10^{8}$	$3.58\times 10^{5}$	$1.09\times 10^{5}$	$6.79\times 10^{6}$	$5.82\times 10^{6}$
</=/>	18/0/12	20/0/10	30/0/0	22/0/8	16/0/14	29/0/1	-

**Table 4:** The 50 Dim Simulation Results of CEC 2017 Benchmark Function.

Function	PSO	BA	SCA	BFGO	ROA	TA	WCO
F1	$9.02\times 10^{6}$	$2.27\times 10^{7}$	$4.87\times 10^{10}$	$1.71\times 10^{7}$	$1.64\times 10^{6}$	$3.45\times 10^{8}$	** $2.60\times 10^{3}$ **
F2	$2.51\times 10^{29}$	** $1.25\times 10^{14}$ **	$4.01\times 10^{67}$	$2.11\times 10^{47}$	$1.06\times 10^{26}$	$2.12\times 10^{58}$	$5.35\times 10^{35}$
F3	** $5.72\times 10^{3}$ **	$4.03\times 10^{4}$	$1.40\times 10^{5}$	$7.17\times 10^{4}$	$1.97\times 10^{4}$	$9.04\times 10^{4}$	$3.95\times 10^{4}$
F4	$7.26\times 10^{2}$	** $5.40\times 10^{2}$ **	$8.24\times 10^{3}$	$6.10\times 10^{2}$	$6.23\times 10^{2}$	$1.06\times 10^{3}$	$6.36\times 10^{2}$
F5	** $7.11\times 10^{2}$ **	$1.15\times 10^{3}$	$1.07\times 10^{3}$	$8.40\times 10^{2}$	$9.95\times 10^{2}$	$9.42\times 10^{2}$	$7.38\times 10^{2}$
F6	$6.53\times 10^{2}$	$6.86\times 10^{2}$	$6.73\times 10^{2}$	$6.56\times 10^{2}$	** $6.46\times 10^{2}$ **	$6.58\times 10^{2}$	$6.59\times 10^{2}$
F7	$1.22\times 10^{3}$	$2.75\times 10^{3}$	$1.70\times 10^{3}$	$1.11\times 10^{3}$	$1.65\times 10^{3}$	$1.60\times 10^{3}$	** $1.09\times 10^{3}$ **
F8	$1.07\times 10^{3}$	$1.42\times 10^{3}$	$1.39\times 10^{3}$	$1.14\times 10^{3}$	$1.52\times 10^{3}$	$1.27\times 10^{3}$	** $1.06\times 10^{3}$ **
F9	$1.16\times 10^{4}$	$3.74\times 10^{4}$	$2.66\times 10^{4}$	$1.19\times 10^{4}$	$2.19\times 10^{4}$	$2.35\times 10^{4}$	** $1.06\times 10^{4}$ **
F10	** $6.87\times 10^{3}$ **	$9.40\times 10^{3}$	$1.50\times 10^{4}$	$8.51\times 10^{3}$	$9.12\times 10^{3}$	$9.40\times 10^{3}$	$8.13\times 10^{3}$
F11	** $1.39\times 10^{3}$ **	$1.45\times 10^{3}$	$8.56\times 10^{3}$	$1.49\times 10^{3}$	$1.42\times 10^{3}$	$2.12\times 10^{3}$	$1.41\times 10^{3}$
F12	** $1.17\times 10^{7}$ **	$4.92\times 10^{7}$	$1.50\times 10^{10}$	$5.39\times 10^{7}$	$1.35\times 10^{7}$	$3.54\times 10^{8}$	$1.69\times 10^{7}$
F13	$9.88\times 10^{4}$	$1.93\times 10^{6}$	$3.61\times 10^{9}$	$3.78\times 10^{5}$	$9.98\times 10^{4}$	$4.25\times 10^{5}$	** $5.84\times 10^{4}$ **
F14	$2.66\times 10^{3}$	$1.09\times 10^{5}$	$3.66\times 10^{6}$	$4.38\times 10^{5}$	$1.92\times 10^{5}$	$3.14\times 10^{5}$	** $1.59\times 10^{3}$ **
F15	$4.12\times 10^{3}$	$6.51\times 10^{5}$	$6.13\times 10^{8}$	$9.17\times 10^{4}$	$2.28\times 10^{5}$	$6.53\times 10^{5}$	** $3.49\times 10^{4}$ **
F16	$3.30\times 10^{3}$	$4.88\times 10^{3}$	$5.75\times 10^{3}$	$4.13\times 10^{3}$	$3.60\times 10^{3}$	$4.58\times 10^{3}$	** $3.16\times 10^{3}$ **
F17	$3.27\times 10^{3}$	$4.02\times 10^{3}$	$4.53\times 10^{3}$	$3.66\times 10^{3}$	$4.01\times 10^{3}$	$3.69\times 10^{3}$	** $3.21\times 10^{3}$ **
F18	** $2.78\times 10^{5}$ **	$1.10\times 10^{6}$	$2.43\times 10^{7}$	$1.12\times 10^{6}$	$3.30\times 10^{6}$	$5.71\times 10^{6}$	$1.32\times 10^{6}$
F19	$3.46\times 10^{3}$	$3.89\times 10^{6}$	$2.98\times 10^{8}$	** $2.69\times 10^{5}$ **	$3.93\times 10^{5}$	$2.89\times 10^{6}$	$8.43\times 10^{5}$
F20	$3.20\times 10^{3}$	$3.98\times 10^{3}$	$4.08\times 10^{3}$	$3.46\times 10^{3}$	$4.04\times 10^{3}$	$3.45\times 10^{3}$	** $3.10\times 10^{3}$ **
F21	$2.60\times 10^{3}$	** $3.00\times 10^{3}$ **	$2.89\times 10^{3}$	$2.70\times 10^{3}$	$2.90\times 10^{3}$	$2.74\times 10^{3}$	** $2.53\times 10^{3}$ **
F22	** $9.40\times 10^{3}$ **	$1.10\times 10^{4}$	$1.66\times 10^{4}$	$1.04\times 10^{4}$	$1.30\times 10^{4}$	$1.14\times 10^{4}$	$9.47\times 10^{3}$
F23	$3.78\times 10^{3}$	$4.07\times 10^{3}$	$3.58\times 10^{3}$	$3.48\times 10^{3}$	$3.68\times 10^{3}$	$3.48\times 10^{3}$	** $3.07\times 10^{3}$ **
F24	$3.82\times 10^{3}$	$4.23\times 10^{3}$	$3.75\times 10^{3}$	$3.67\times 10^{3}$	$3.61\times 10^{3}$	$3.69\times 10^{3}$	** $3.20\times 10^{3}$ **
F25	$3.12\times 10^{3}$	$3.00\times 10^{3}$	$7.24\times 10^{3}$	$3.07\times 10^{3}$	$3.11\times 10^{3}$	$3.35\times 10^{3}$	$3.13\times 10^{3}$
F26	$1.07\times 10^{4}$	$1.46\times 10^{4}$	$1.25\times 10^{4}$	$9.25\times 10^{3}$	$1.20\times 10^{4}$	$1.10\times 10^{4}$	** $6.78\times 10^{3}$ **
F27	$4.81\times 10^{3}$	$3.36\times 10^{3}$	$4.58\times 10^{3}$	** $3.21\times 10^{3}$ **	$4.01\times 10^{3}$	$4.30\times 10^{3}$	$4.01\times 10^{3}$
F28	$3.38\times 10^{3}$	$3.30\times 10^{3}$	$7.40\times 10^{3}$	$3.39\times 10^{3}$	** $3.28\times 10^{3}$ **	$3.97\times 10^{3}$	$3.45\times 10^{3}$
F29	$5.48\times 10^{3}$	$6.44\times 10^{3}$	$7.65\times 10^{3}$	$5.59\times 10^{3}$	$8.23\times 10^{3}$	$6.79\times 10^{3}$	** $5.24\times 10^{3}$ **
F30	$8.95\times 10^{5}$	$4.03\times 10^{7}$	$7.33\times 10^{8}$	** $1.96\times 10^{6}$ **	$3.30\times 10^{7}$	$1.32\times 10^{8}$	$1.10\times 10^{7}$
</=/>	17/0/13	25/0/5	30/0/0	23/0/7	21/1/8	29/0/1	-

**Table 5:** Position and velocity of anchor nodes.

Anchor Node	m			m/s		
	$x_{i}$	$y_{i}$	$z_{i}$	${\dot{x}}_{i}$	${\dot{y}}_{i}$	${\dot{z}}_{i}$
1	300	100	150	30	−20	20
2	400	150	100	−30	10	20
3	300	500	200	10	−20	10
4	350	200	100	10	20	30
5	−100	−100	−100	−10	10	10

**Table 6:** The optimization results for time design optimization.

Algorithm	TSWLS	PSO	BA	WCO
Time (s)	72.819125	82.471092	80.814939	61.347426

## Data Availability

Not applicable.
